# Alleged Presence of the Plague in the Metropolis

**Published:** 1847-09

**Authors:** 


*Alleged Presence of the Plague in the Metropolis.*—Dr. Tweedie has addressed a letter to the Times, in reference to the prevalence of typhus fever under a severe form in the metropolis. After stating that the admissions to the Fever Hospital during the present year have been but little above the average of the last five years, he says:—"In regard to the character of the cases received since January last, it is important to observe, that a very large majority have not been cases of typhus fever, but of inflammation of internal organs, more especially of the lungs, which every medical practitioner knows to be invariably accompanied with more or less feverish disturbance of the system. Indeed, for some time past, the proportion of cases of genuine typhus fever has been unusually small, much smaller than, from the late scarcity of provisions, and consequently inadequate supply of food, could have been anticipated.

"London, therefore, affords at this time a striking contrast to many of the large provincial towns, in which typhus fever, from causes which it is unnecessary to advert to at present, prevails to such an alarming extent.

"And lastly, with respect to the tendency to inflammation of the glands of the face and neck—the circumstance on which the apparent connexion with plague is founded—it may be remarked, that this symptom is occasionally observed in very mild cases of fever, but that it has not been a very frequent occurrence lately, is evident from the fact that it has been observed in only five of 452 cases received into the Fever Hospital since January last; and that of these five only two have died, the fatal event in one being the result, not of the glandular inflammation, but of old standing diseases of the chest.

"From these facts I think it may be deduced,—1st. That typhus fever is not at the present time alarmingly prevalent in London; 2d, that in its character it bears no analogy to the plague; and 3d, that the inhabitants of London have great cause of thankfulness that as yet the metropolis has been visited with a much less proportionate amount of epidemic fever than any other city in the kingdom."

The mortality from typhus fever is at present much above the average! the deaths last week were 52 to a spring average of 34.—*London Medical Gazette.*

